# Metagenomic Characterization of the Viral Community of the South Scotia Ridge

**DOI:** 10.3390/v11020095

**Published:** 2019-01-24

**Authors:** Qingwei Yang, Chen Gao, Yong Jiang, Min Wang, Xinhao Zhou, Hongbing Shao, Zheng Gong, Andrew McMinn

**Affiliations:** 1 College of Marine Life Sciences, Ocean University of China, Qingdao 266003, China; foodyqw_12@163.com (Q.Y.); gaochen@stu.ouc.edu.cn (C.G.); piglet.naruto@gmail.com (X.Z.); hbshao@ouc.edu.cn (H.S.); gongzheng-1991@163.com (Z.G.); yangqingwei@stu.ouc.edu.cn (A.M.); 2 Key Lab of Polar Oceanography and Global Ocean Change, Ocean University of China, Qingdao 266003, China; 3 Institute of Evolution and Marine Biodiversity, Ocean University of China, Qingdao 266003, China; 4 Institute for Marine and Antarctic Studies, University of Tasmania, Hobart, Tasmania 7001, Australia

**Keywords:** virus, South Scotia Ridge, viral community, diversity, Pgvv-like

## Abstract

Viruses are the most abundant biological entities in aquatic ecosystems and harbor an enormous amount of genetic diversity. Whereas their influence on marine ecosystems is widely acknowledged, current information about their diversity remains limited. We conducted a viral metagenomic analysis of water samples collected during the austral summer of 2016 from the South Scotia Ridge (SSR), near the Antarctic Peninsula. The taxonomic composition and diversity of the viral communities were investigated, and a functional assessment of the sequences was performed. Phylotypic analysis showed that most viruses belonged to the order *Caudovirales*, especially the family *Podoviridae* (41.92–48.7%), which is similar to the situation in the Pacific Ocean. Functional analysis revealed a relatively high frequency of phage-associated and metabolism genes. Phylogenetic analyses of phage TerL and Capsid_NCLDV (nucleocytoplasmic large DNA viruses) marker genes indicated that many sequences associated with *Caudovirales* and NCLDV were novel and distinct from known phage genomes. High *Phaeocystis globosa* virus virophage (Pgvv) signatures were found and complete and partial Pgvv-like were obtained, which influence host–virus interactions. Our study expands existing knowledge of viral communities and their diversities from the Antarctic region and provides basic data for further exploring polar microbiomes.

## 1. Introduction

Viruses exist wherever life is found, including in extreme locations such as the deep ocean and polar areas. Arguably, viruses are by far the most numerous, genetically diverse, and pervasive biological entities on earth [1,2], including in aquatic ecosystems [3,4]. They are critical mortality agents of both eukaryotes and prokaryotes, affecting the abundance and diversity of microbial communities as well as global biogeochemical processes and energy fluxes, by causing lysis of a large proportion of both autotrophic and heterotrophic prokaryotes, shunting nutrients between particulate and dissolved phases [5,6,7,8,9,10], and modifying the efficiency of the carbon pump [11]. The genetic diversity of bacteria and protists was shaped by virus-mediated horizontal gene transfer, allowing viral genes to spread widely [4,12].

Both the ecology of Antarctic prokaryotes and protists [13,14,15,16,17,18] and the major role of viruses in prokaryotic and eukaryotic phytoplankton mortality [19,20,21,22] have been well studied. However, due to the geographical isolation and difficulty of accessing the Antarctic and Sub-Antarctic and the culturing viral hosts, an understanding of virus diversity and viral community structures in these regions is still lacking. There are comparatively few studies based on culture-independent methods, such as metagenomics and single-cell genomics, on the DNA and RNA of viral communities in Antarctica, although there are a few from freshwater habitats [23,24,25,26,27], the Southern Ocean close to the Western Antarctic Peninsula [28], and sediment soils [29,30]. These studies all identified a high viral biodiversity in these Antarctic ecosystems. However, despite the virome diversity information derived from these special habitats in the Antarctic, few studies have been completed in the open sea near the Antarctic Peninsula.

In this study, we conducted an analysis of viromes from three South Scotia Ridge (SSR) seawater samples including two from the surface and one from the bottom (water depth = 521 m) in an area influenced by Antarctic Circumpolar Current flow (ACC) [31]. The taxonomic composition of these viromes and the dominant viral species were identified and compared with viromes from other areas and habitats. A phylogenetic and/or genomic analysis of the representatives was undertaken.

## 2. Methods and Materials

### 2.1. Sample Collection and Sequencing

Seawater samples, including two surface waters and one from bottom water, at 5 m above the sediment–water interface (Table S1), were collected during the austral summer (December 2016) from two sites (D39 close to the edge of the Powell Basin and DA4 near the Clarence and Elephant Islands, Figure S1) on the southern flank of the SSR. Seawater temperature and salinity were recorded with a CTD profiler (SBE9/11 plus V5.2, Sea-Bird Inc., Newport, RI, USA). The temperatures of the seawater samples ranged from −0.04 to −0.57 °C and the salinity from 34.37‰ to 34.57‰ (Figure S1, Table S1). Water for biological and chemical analysis was collected with Niskin bottles attached to the CTD profiler and was prefiltered with 20-μm mesh to remove large particles.

The virome samples were processed immediately, according to the process used by Sun et al. [32]. Briefly, the samples were sequentially filtered through 3-μm and 0.22-μm pore size filters to remove any microorganisms, and then a two-step tangential flow filtration (TFF) with a 50-kDa cartridge (Pellicon^®^ XL Cassette, Biomax^®^ 50 kDa; polyethersulfone, Millipore Corporation, Billerica, MA, USA) was used to concentrate the viruses to a final volume of ca. 50 mL and stored at −80 °C. The samples were further concentrated by polyethylene glycol (PEG-8000) precipitation (10% *w*/*v*) and incubated at 4 °C overnight. The concentrate was then centrifuged at 8000× *g* for 80 min at 4 °C and suspended in 200 µL of SM buffer (0.05 M TRIS, 0.1 M NaCl, 0.008 M MgSO_4_, 0.01% (*w/v*) gelatin pH 7.5). Finally, DNA was extracted using the phenol/chloroform/isoamyl method and precipitated with ethanol without random amplification. High-throughput sequencing was performed by Novogene (Beijing, China) using Illumina Hiseq X ten (Paired End sequencing, 2 × 150 bp).

### 2.2. Virome Composition Analysis

The paired-end reads were quality trimmed by adopting the following conditions: (1) they contained more than 10% N, (2) had an adapter, and (3) were of low quality (70% read length, Q ≤ 30, and 80% read length, Q ≤ 20). All clean sequence data with quality-controlled were submitted to the National Center for Biotechnology Information (NCBI) Sequence Read Archive (SRA) under the following accession numbers: PRJNA505984 [33].

In order to avoid chimeras, SSR virome sequences were analyzed without assembly and queried by Diamond [34] against the NCBI non-redundant (nr) protein database [35] and the RefSeq complete viral genomes protein references (viral RefSeq) database [36], setting a maximum *E*-value of 10^−3^. Taxonomic identification was assigned based on best similarities and the relative taxonomy was normalized against complete viral genome length and sequencing depth which is the number of reads annotated to the virus divided by the viral complete length and the total number of reads sequenced in this sample.

### 2.3. Virome Comparison Analysis

Twenty previously published viromes taxonomic compositions, with the same maximum E-value based on read number, were selected from MetaVir to compare with this study [37]. These were obtained from a variety of habitats, including six temperate freshwater lakes (Lake Bourget and Lake Pavin [38], Antarctic lakes [23], Lough Neagh [39], Tilapia Channel [40]), nine seawater sites from the eastern tropical South Pacific oxygen minimum zones (ETSP-OMZ) [41], the Indian Ocean [42], the high salinity Jiulong River Estuary [43], Dunk Island, Fitzroy Island, LA26S (near Vancouver Island in British Columbia, Canada), M1CS (Monterey Bay, CA, USA) of the Pacific Ocean Virome (POV) [44], the Arctic Ocean, and Sargasso Sea (SAR) [45], three deep-sea surface sediment samples (Arctic Ocean, Black Sea and Mediterranean Sea [46]), soil, and hypolithon [47] (Table 3). The relative taxonomic composition of each community was normalized as described above. The similarity search algorithm BLAST was performed on the three SSR viromes against the 20 viromes obtained in MetaVir. The taxonomic composition distance matrix, based on relative abundances, was used in the non-metric multidimensional scaling(nMDS) analysis to plot viromes (the metaMDS function with a Bray–Curtis dissimilarity index using the VEGAN package in R software [48]) and a PERMANOVA (Permutational multivariate analysis of variance) test (*p*-test) was also performed.

In order to completely compare viromes rather than only their small known fraction, a qualitative comparison of viromes based on sequence similarity (tBLASTX comparison) was computed as described in MetaVir [37]. Briefly, we completed tBLASTX searches of the sub-sample set of sequences (that is, 50,000 sequences of 100 bp were randomly extracted from each entire virome library) from every single library versus all other subsamples. A similarity score between virome A and virome B was then computed as the sum of the top High Scoring Pairs (HSPs) scores of virome A reads against virome B reads (*E*-value < 10^−5^). Finally, the resulting score matrix (i.e., similarity scores for all virome pairs) was used in the nMDS analysis to plot viromes as described above.

### 2.4. Metagenomic Assembly and Function Analysis

SSR virome assemblies were performed via a random subsampling approach as previously described [49]. They were designed to obtain as the longest possible contigs by reducing the microdiversity within the samples [50,51]. Briefly, the assembling strategy was based on random selection of a subset of the reads: 1% (75×), 5% (50×), 10% (50×), 25% (25×), 75% (25×), and 100% (1×) from each sample and then assembling these subsets individually with IDBA_UD (v 1.1.2) [52] using the default parameters. We combined contigs derived from all the assemblies of the same samples and removed those <500 bp. To this end, contigs were clustered at 90% global average nucleotide identity with cd-hit-est (v 4.7, options: -c 0.9 -n 8) [53]. The relative abundance of each non-redundancy (nr) contigs was determined based on the mapping of the quality-filtered reads to the contigs, computed with bowtie2 (v 2.3.3.1) [54] and SAMtools [55], using the default parameters (the total length of reads mapping to the contig divided by the contig length). Then, the nr contigs were uploaded to the IMG system [56,57] and analyzed with the standard operating procedure of the DOE-JGI Metagenome Annotation Pipeline (MAP v.4) [58]. Finally, the IMG genomes 3300028548, 3300028550, and 3300028925 were obtained. The functional content was further characterized using MG-RAST [59] (with MG-RAST accession number 4808192.3, 4808195.3 and 4808193.3 respectively), an online metagenome annotation service [60], which was used to compare data to the SEED Subsystems database using a maximum *E*-value of 10^−5^, a minimum identity of 60%, and a minimum alignment length of 15.

### 2.5. Phylogenetic Analysis

Two double-stranded DNA (dsDNA) markers were present: the phage terminase large-subunit domain (*TerL*), which was present in phages of the order *Caudovirales* (Terminase_6, PF03237), and the major capsid protein (*MCP*) gene, which was present in large eukaryotic DNA viruses (Capsid_NCLDV, PF04451). Both of these were used to construct the phylogenetic trees and the TerL sequences were dereplicated at the 97% nucleotide level using cd-hit [53]. These markers from the SSR virome genes were screened by the DOE-JGI Metagenome Annotation Pipeline and compared to the viral RefSeq database using BLASTP (*E*-value < 10^−5^) to recruit relevant reference sequences. All sequences were aligned at the amino acid level using MUSCLE [61] (using default parameters), manually inspected and trimmed as necessary, and both maximum likelihood (ML) trees (MCP and TerL) with 1000 bootstraps were constructed with the program FastTree (v2.1.10) [62] using a JTT + CAT model and an estimation of the gamma parameter. Finally, the data were visualized and displayed using iTOL (Interactive Tree of Life) [63].

### 2.6. Genomic Comparison

The *Phaeocystis globosa* virus virophage (Pgvv)-like genomes were annotated with RAST and predicted open reading frames (ORFs) were searched against the NCBI reference viral protein (taxid:10239) with online BLASTP [64]. The partial functional annotations of the Pgvv reference sequence was obtained from Yutin et al. [65]. Visualization of the genomes’ map comparisons was completed EasyFig [66].

## 3. Results

### 3.1. Overview of SSR Viromes

**Figure 1:**
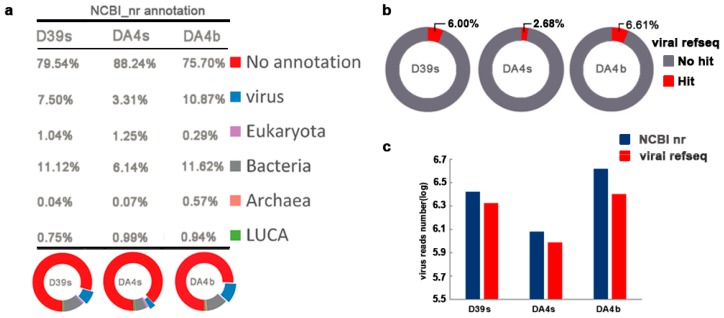
Taxonomic assignment of metagenomic reads (**a**) Percentage of the sequence reads classified by the taxonomic grouping based on BLASTX similarity search with NCBI nr database (*E*-value < 10^−3^). Sequences with no hits with *E*-value > 10^−3^ were regarded as unidentified reads (“no annotation” category in the table and red in the pie graphs). “LUCA” (last universal common ancestor) (green) denotes reads that could not unambiguously be assigned to a domain of life. (**b**) Taxonomic assignment of metagenomic reads based on BLASTX similarity search with viral RefSeq database (*E*-value < 10^−3^). (**c**) Comparison of the annotation results of NCBI nr and viral RefSeq.

After extraction, a total of 129,710,606 paired-end 150 bp sequences, with 109,923,264 (84.75%) reads passing the quality screening (Table 1), were obtained. The best BLAST Hit (*E*-value < 10^−3^) affiliations of unassembled high-quality reads from the three data sets are consistent with viral metagenomes published so far, as more than three-quarters of the reads (75.7–88.24%) did not show any significant sequence similarity to current NCBI nr data (Figure 1a). According to the NCBI nr and viral RefSeq annotation, the reads classified as viruses were 3.31–10.87% and 2.68–6.61% respectively (Figure 1b). A comparison of the annotation results of NCBI nr and viral RefSeq, showed that the virus sequences annotated with virus in the NCBI nr database were more abundant than those in viral RefSeq (Figure 1c), indicating that a certain proportion of sequences belong to an unidentified virus that viral RefSeq excluded, such as the uncultured Mediterranean phage uvMED.

### 3.2. Taxonomic Diversity Analysis

The BLAST data results (against viral RefSeq) of the virome composition were visualized using the Krona tool (Figures S2–S5) [67], which showed that, as expected, the majority of viral reads (93.69–95.16%) with significant hits belonged to double-stranded DNA (dsDNA) viruses with no RNA stage. These were largely comprised of members of the *Caudovirales* comprising the families *Podoviridae*, *Siphoviridae*, and *Myoviridae*, with similarities to single-stranded DNA (ssDNA) viruses also observed (Table 2 and Table 3). *Podoviridae* sequences (41.92–48.7%) were the most abundant in all three viromes, followed by *Myoviridae* (22.92–29.46%) and *Siphoviridae* (11.92–14.08%). Viruses from *Phycodnaviridae* (infecting algae) and *Mimiviridae* (infecting amoebas and algae) were more abundant in surface waters than the bottom water (D39s: 3.57% and 0.16%, DA4s: 2.22table% and 0.22%, DA4b: 1.32% and 0.10%, respectively). There was a significant proportion of virophages that prey on phycodnaviruses in the surface water samples: approximately 2% in D39s (Table 2). The top 10 most abundant viral species (Figure S6) included *Puniceispirillum* phage HMO-2011 [68] (*Podoviridae*, circular genome), which infects a bacterium of the SAR116 clade and was the most abundant in the SSR virome (18.50–25.75%), accounting for ~25% in DA4s, and Pelagibacter phage HTVC008M [69] (*Myoviridae*, linear), a T4-like myovirus infecting a SAR11 bacteriophage, which was the second most abundant (8.6–11.11%).

### 3.3. Comparison with Other Viromes

**Figure 2:**
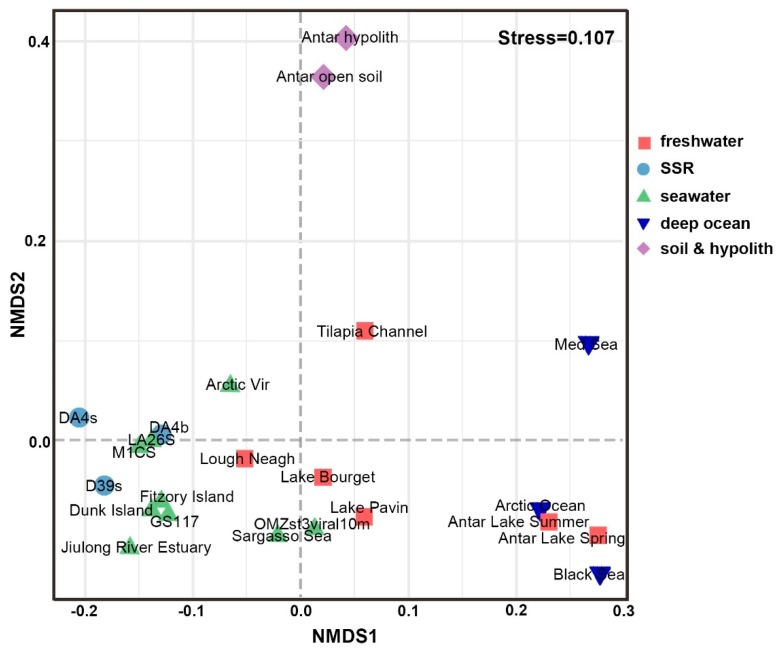
Comparison viromes between the SSR area and other environmental viromes depending on BLAST-based comparison. Twenty environmental viromes were available on MetaVir2, obtained from different habitats including freshwater, seawater, deep-sea surface sediments, soil, and hypolith. Bray–Curtis dissimilarity matrices of BLAST hits were calculated from virome data and used to represent the relative distances between individual viromes (stress value 10.7%).

**Figure 3:**
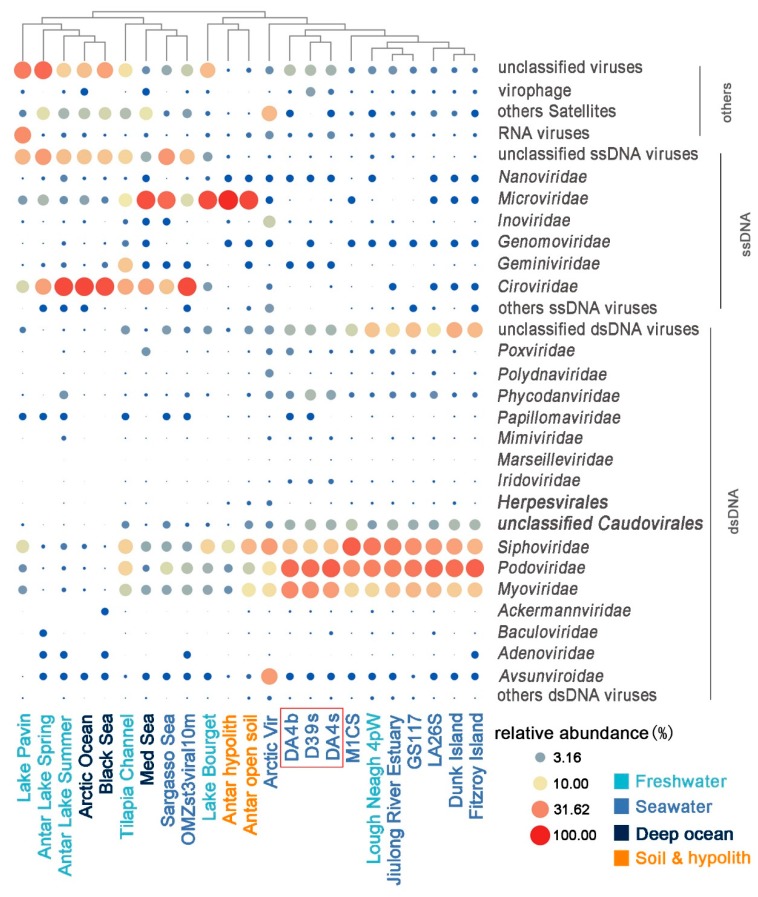
The relative abundance of viral sequences (normalized with genome length) mostly at the family level in each different habitat virome. Point size indicates the value of relative abundance percentage.

To compare viromes from the present study with previously published data sets, 20 viromes from different habitats were selected from MetaVir (see Materials and Methods for details). The result showed that the three SSR viromes were most closely related to ocean surface samples, except for the samples from ETSP-OMZ and SAR (Figure 2 and Figure S5) (*p* < 0.001). At the ocean surface, virome composition at the family level was dominated by the *Caudovirales* (*Myoviridae*, *Siphoviridae*, and *Podoviridae*), which collectively contributed 43.74–92.03% of the genomes. Viromes from within special habitats, including deep-ocean surface sediments, ETSP-OMZs, Antarctic freshwater, soil, and hypolithon, are dominated by *Ciroviridae* and *Microviridae,* members of ssDNA viruses, which contributed 25.45–88.45% of the genomes (Figure 3, Table 3). Less than 5% of these viromes’ sequences showed any similarity (*E*-value < 10^−3^) to the SSR viromes (Viromes were highlighted in bold shown in Table 4).

### 3.4. Contigs and Function Analysis

As the contigs assembled by the random subsampling approach could still contain redundant sequences derived from the same (or closely related) populations contigs derived from the same population were merged into clusters with 90% global average nucleotide identity using cd-hit-est. This resulted in 145,023 (D39s), 135,910 (DA4s), and 234,648 (DA4b) non-redundant genome fragments (>500 bp) (Table 1). Of these, 43.32% (D39s), 35.07% (DA4s), and 55.49% (DA4b) quality-filtered reads were assigned to nr contigs.

**Figure 4:**
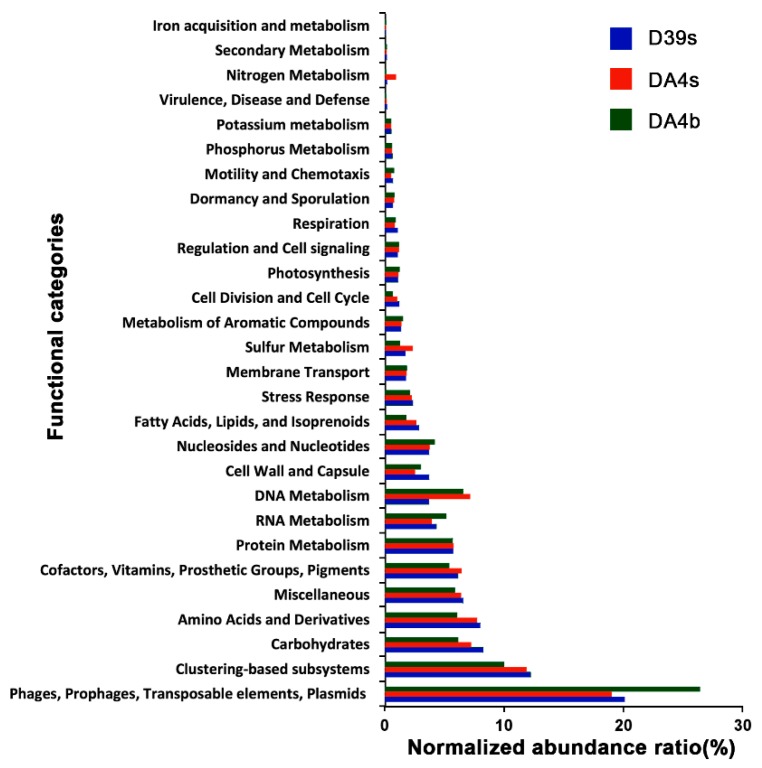
Composition of predicted functional genes of the SSR contigs. The Coding Sequences (CDSs) were compared with the SEED database using subsystems in MG-RAST. The metabolic categorization is based on the sequences Best BLAST Hits in the SEED database curated subsystems (*E*-value < 10^−5^).

The putative functions of the annotated ORFs from the nr contigs dataset were predicted using MG-RAST, which assigns sequences to metabolic categories based on their Best BLAST Hit against the SEED database (*E*-value < 10^−5^). Using the subsystems approach, nearly 25% (17.54–26.46%) of the annotated proteins fell into ‘Phage, Prophage, Transposable elements, or Plasmids’ (Figure 4). Phage structural, integration/excision, and DNA metabolism-related proteins were most commonly identified and 10–11.96% of them were classified into “Clustering-based subsystems”, with phage endolysin commonly found in this category. The other SEED functional annotation categories showed that the metabolism of amino acids, carbohydrates, cofactors, vitamins, proteins, RNA, DNA, and nucleosides/nucleotides were the dominant annotations. In these categories, many proteins, such as DNA polymerases and helicase, could be phage-related (or of possible cellular origin). These hits were also found in the Pfam and COGs databases (see IMG system), with ‘Replication, recombination and repair’ being the most common protein categories identified.

### 3.5. Phylogenetic Tree Analysis

#### 3.5.1. Terminase Phylogeny

**Figure 5:**
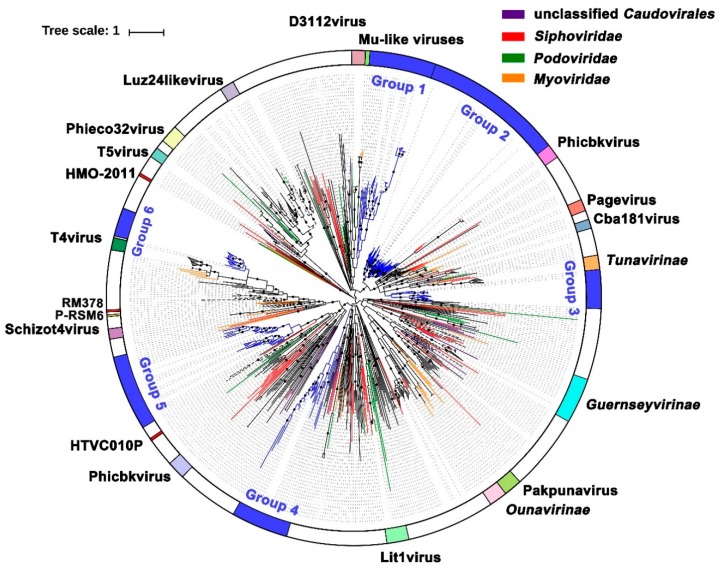
Terminase phylogeny. A maximum-likelihood phylogenetic tree of *Caudovirales* terminase large-subunit domains (PF03237) is shown (1000 iterations, JTT + G model). Only bootstrap values >50% are indicated at the nodes of the tree, and bootstrap scores greater than 90% are indicated with a black dot. Average branch length distance of leaves less than 0.4 were collapsed and are shown as triangles. Reference sequences are marked (see color legend at the top). Abbreviations are as follows. HMO-2011: *Puniceispirillum* phage HMO-2011; HTVC010P: *Pelagibacter* phage HTVC010P; RM378: *Rhodothermus* phage RM378; P-RSM6: *Cyanophage* P-RSM6. The black dotted line, solid line, and bright blue line indicate the sequences obtained from this study.

The ML phylogenetic analysis of the phage large terminase subunits identified in this work is shown in Figure 5. The topology of the phylogenetic tree clearly shows that the majority of the SSR viromes’ TerL amino acid sequences were widely distributed among the *Myo-*, *Sipho-,* and *Podoviridae*. Among them, several branches (black dotted line) were moderately related to known members of the *Guernseyvirinae* family, *T5virus*, *Luz24likevirus*, and *T4virus* genera, and a few sequences were relatively closely related to those of known cultured representatives, including *Pelagibacter* phage HTVC010P, *Rhodothermus* phage RM378, and *Cyanophage* P-RSM6. Most sequences, however, were phylogenetically distant to known complete phage genomes (black solid lines). Notably, six groups (bright blue solid line) did not cluster with any known species and formed novel phylogenetic clusters. This separation is supported by high bootstrap values, which highlight the important but uncharacterized diversity of the *Caudovirales* in SSR.

#### 3.5.2. Capsid_NCLDV Phylogeny

**Figure 6:**
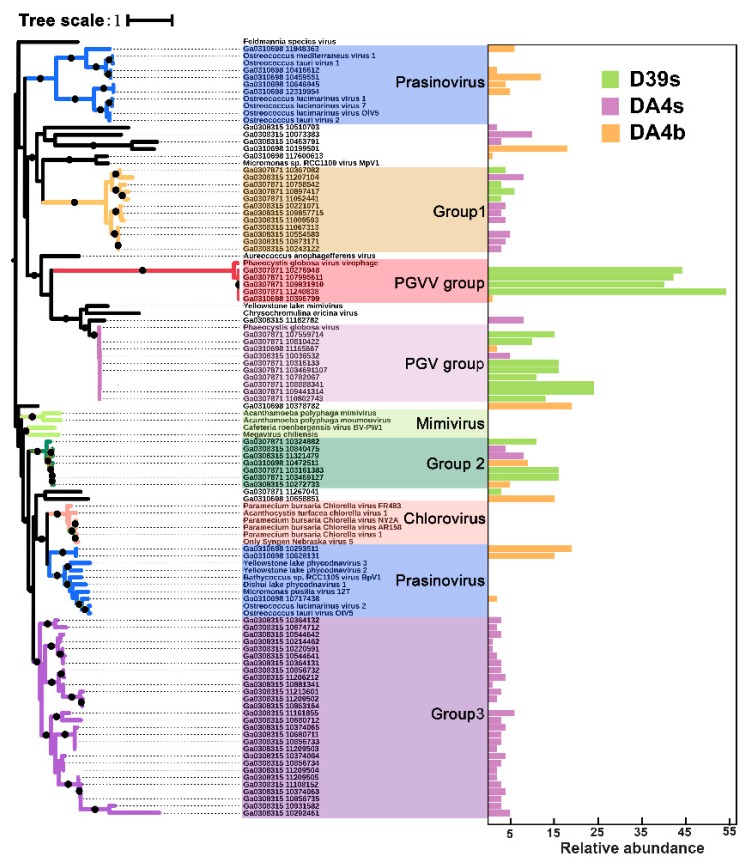
Capsid_NCLDV phylogeny. A maximum-likelihood phylogenetic tree drawn from the capsid_NCLDV (PF04451) and six virophage putative major capsid (MCP) protein multiple alignment is shown (1000 iterations, JTT + G model). Bootstrap scores greater than 90% are marked with black dots. Each MCP is associated with an abundance profile (right) that displays the relative abundance of the contig across the three SSR viromes (based on normalized coverage).

An ML phylogenetic tree, based on the MCP, which includes a group of putative MCP of Pgvv-like infected *Phaeocystis globosa* virus (Pgv), is shown in Figure 6. The MCP tree shows that several sequences from the SSR viromes are closely related to known NCLDV—mainly those belonging to Phycodnaviruses, and these can be classified into Prasinovirus, Pgv, and Pgvv. The three clades differed from Phycodnaviruses and Mimivirus MCPs and formed three distinct groups with the well-supported clades. One of the clades, marked as Group3, was only found in the surface ocean of DA4 station. The Pgv group, which included five new Pgvv-like MCPs, was distantly related to the Pgv group and had a higher relative abundance in the surface samples than the bottom samples.

### 3.6. Novel Pgvv Group

**Figure 7:**
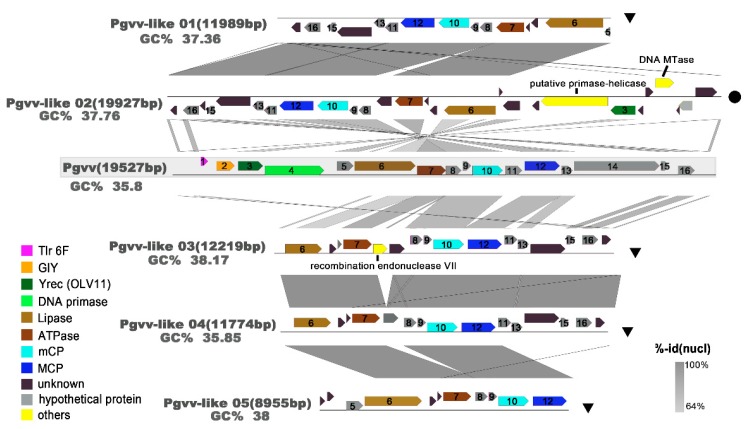
Overview of genomic synteny and similarities between Pgvv-like group. The complete Pgvv reference is covered by a grey shadow. A color scale for percent identity (nucleic) is shown at the bottom right. The name, percent GC content (GC%), and length for each genome are indicated. Genes are colored according to their functional affiliation. Tlr 6F: Toll-like receptor 6 family; GIY: GIY-YIG family nuclease; MCP: major capsid protein; mCP: minor capsid protein; and Yrec (OLV11): OLV11-like tyrosine recombinase.

From the MCP phylogenetic tree, one distinct group of virophages was defined for which there is one known related virophage genome. However, this group still different from the known Pgvv. An alignment of Pgvv-like group genomes is shown in Figure 7. The Pgvv-like group genomes appear to have a relatively high GC content (37.36–38.17%), which was expected as the GC content of the Pgvv-like 04 genome (GC, 35.85%) was similar to Pgvv (GC, 35.8%). All virophages share four homologous proteins or domains: (1) packaging ATPase (ATPase), (2) lipase, (3) major capsid protein (MCP), (4) minor capsid protein (mCP). In addition, Pgvv-like 02 also contains the OLV11-like tyrosine recombinase (*Yrec*) gene, which is distantly related to the OLV11-like family [65]. Three genes with functional annotation (shown in yellow), which were absent in the Pgvv genome, were carried by the Pgvv-like sequences, including putative primase-helicase and DNA methyltransferase genes in the Pgvv-like 02 and recombination endonuclease VII gene in Pgvv-like 04. These characteristics further indicate that these viruses may belong to a new Pgvv-like group.

## 4. Discussion

Marine viral communities are still largely undescribed and many basic features, such as their global ocean distribution and their actual genetic and species richness, remain unknown [4,11,70]. With the advent of metagenomic methods, an association with high-depth sequencing, and meta-analyses of bioinformatics, an increasing number of studies have been conducted [71,72]. So far, only a few of these have focused on viral communities from the Antarctic region and most of these are from unusual habitats, such as freshwater lakes [23], hydrothermal vents [73], and soils [30].The important role of marine dsDNA viral communities viruses (that is, those capable of both lysogeny and lytic replication) in the Western Antarctic Peninsula has recently been demonstrated [28]. Also, the major differences in viral community composition between the subtropical Indian and the Southern Oceans have been identified [74].

The number of reads identified as either bacteria or eukaryote was similar to that reported in viral metagenomes of other environments [70,75]. In addition, the relatively low number of rRNA and tRNA genes (<1%) matching sequences (Table S2) indicates a certain degree of bacterial and eukaryotic contamination of the metagenomes; this has previously been reported to occur with TFF-based concentration methods [76]. One possible reason is that bacterial genes can be packaged into generalized transduced phage particles [77,78]. The bacterial-like sequences might have originated from excised prophages, mistakenly annotated as bacterial, and/or from genes of bacterial origins that were transferred to their phages [75]. Another problem is that cd-hit-est does not de-replicate full circular genomes that are linearized at different assembly sites, leading to circular viruses of the same source potentially merging into different clusters, which does not affect the reads-based taxonomic identification.

BLAST searches showed that more than 75% of the sequences before assembly did not have homologs in current sequence databases. This is consistent with the results of previously published viral metagenomic projects [44,74,79,80]. The SSR viromes were mostly dominated by *Caudovirales*, including Myoviruses, Siphoviruses, and Podoviruses, which are the dominant viral types recovered during metagenomic analyses of most marine environments [4,79]. In the three SSR viromes investigated here, the largest number of reads (>40%) were related to podoviruses and ~13% of reads were of siphoviruses (viruses that infect photosynthetic bacteria such as *Prochlorococcus* and *Synechococcus*) (in bold, Table S3). Consistent with a previous investigation [68,81], *Puniceispirillum* phage HMO-2011, which infects *Candidatus Puniceispirillum marinum* strain IMCC1322 of the SAR116 clade, and the *Pelagibacter* phage group (HTVC008M, HTVC010P, HTVC011P, and HTVC019P), infects SAR11 populations were widespread and most abundant in the SSR. Both SAR11 and SAR 116 clades play important roles in oceanic dimethylsulfide (DMS) production and biogeochemical sulfur cycles, especially via bacteria-mediated dimethylsulfoniopropionate (DMSP) degradation [82,83]. Marine viruses are likely to indirectly influence the global sulfur cycle by mediating the death of both hosts. Interestingly, Pgv is the tenth most abundant viral species in the SSR region (2.53% in D93s), infecting the temperate algal species *Phaeocystis globosa* [84]. In the Antarctic, however, the most abundant *Phaeocystis* species is *P. antarctica* [85], but a *P. antarctica*-specific virus has not yet been isolated or identified, which may suggest a high genome similarity between *P. antarctica* virus and Pgv. Compared with the surface viromes, a relatively smaller number of *Phycodnaviridae* and their virophage were still identified in the bottom virome where eukaryotic algae cannot photosynthesize. The origin and activity of these viruses needs further study.

Despite being in a cold marine environment with an average temperature below 0 °C, the SSR viral community had a similar structure to those found in the Pacific Ocean. However, there were still significant differences in nucleic acid levels. Is likely that the genotype of many viruses changed, allowing them to infect psychrophiles and thus evolve into new viral groups. The previously studied viromes from deep-ocean surface sediments, ETSP-OMZs, Antarctic freshwater, soil and hypolithon, in which ssDNA viruses played dominated roles, were clearly different from those of the SSR. However, all of those viromes, except those from the deep-ocean surface sediment, were amplified using multiple displacement amplification (MDA) with phi29 polymerase. In these genomes of the ssDNA, viruses were selectively amplified [86,87], leading to an overestimation of the role of ssDNA viruses. Although existence bias from MDA in these studies and the prevalence of *Caudovirales* sequences has been observed in most marine viromes, previously published research on global morphological analysis of marine viruses, conducted by the Tara Oceans Expedition, showed that non-tailed viruses (largely ssDNA and RNA) numerically dominate the upper oceans [88], and small, non-tailed viruses were undoubtedly underestimated in the SSR region.

The deep sequencing method, combined with a random subsampling assembly approach, enable obtaining a nearly complete viral genome and undertaking phylogenetic analyses on marker genes. Analysis of the major viral groups found in the SSR viromes showed broad diversity with many previously unknown virotypes. The terminase gene, which is responsible for DNA recognition and initiation of DNA packaging, is an essential component of all head-tail phages (*Caudovirales*), as it encodes the molecular movements that translocate DNA into empty capsids [89]. There is a large diversity of terminases that can be used to resolve different Caudoviruses groups [90]. The NCLDV comprises a monophyletic group of viruses infecting both animals and a diverse range of unicellular eukaryotes, including the *Phycodna-*, *Mimi-*, *Asco-*, *Asfar-*, *Irido-*, and *Poxviridae* families. The MCP of NCLDV (capsid_NCLDV), a redox protein that encodes complex DNA replication and transcription systems and involved in the formation of disulfide bond in virion membrane proteins, is relatively conserved among NCLDVs evolution [91,92,93]. Using phylogenetic trees based on these two viral marker genes (TerL and MCP), a high diversity among *Caudovirales* and NCLDV was identified. In a single habitat, the high diversity of viruses not only expressed in genotypes, but also in morphological and biological properties such as plaque morphology, originated from a broad range of hosts and propagation temperatures [73]. A high proportion of TerL sequences was distributed both far from the reference and far from each other, highlighting both the richness of *Caudovirales* in the SSR communities and the absence of closely-related reference sequences. In addition, some SSR virome sequences appear to have formed a new clade (Group 6) related to the T4 viruses, one of the best described *Caudovirales* families.

The topology of the MCP tree and genomic comparisons strongly suggest that the five putative virophage genomes are more closely related to the Pgvv than to other NCDLV families, including the Pgvv host. The Pgvv-like group also has a high relative abundance. The Lotka–Volterra simulation demonstrated that virophages promote secondary production through the microbial loop by reducing overall mortality of the algal cell after a bloom and increasing the frequency of blooms during the summer [20]. According to the above model, it can be inferred that the Pgvv-like group plays a previously unrecognized role in regulating virus-host interactions in the SSR area during summer.

## 5. Conclusions

Analysis of the SSR viromes has shown that novel, oceanic-related viromes are present. A high proportion of sequence reads was classified as unknown, with only 3.31–10.87% having known virus counterparts. Among these, members of the order *Caudovirales* were most abundant. This pattern is consistent with previously described viromes from the Pacific Ocean as well as from a range of different biomes. The diversity of the *Caudovirales* and NCLDV in the SSR viromes is high, suggesting that viral diversity is high in gelid environments. However, the abundance and diversity of ssDNA and RNA viruses require further research. The strong signatures of Pgvv found in the SSR may indicate that virophages play an important role in regulating virus-host interactions.

## Figures and Tables

**Table 1:** The remaining number of quality-controlled reads and non-redundancy contigs. Q20: quality score of 20; Q30: quality score of 30.

Process		SSR Viromes		
		D39s	DA4s	DA4b
**Quality control**	Raw reads	4,2547,324	43,791,908	43,371,374
	Cut adaptor	40,670,620 (95.59%)	41,883,752 (95.64%)	41,353,540 (95.35%)
	Q20 >20%	35,357,306 (83.10%)	37,567,224 (85.79%)	38,397,082 (88.53%)
	Q30 >30%	35,334,370 (83.05%)	36,385,270 (83.09%)	38,203,624 (88.08%)
**Assemble**	All assembled contigs (>500 bp)	2,418,081	3,699,559	1,693,019
	Non-redundancy contigs	145,023	135,910	234,648
**Mapping**	Mapped reads	15,307,526 (43.32%)	12,760,519 (35.07%)	21,197,289 (55.49%)

**Table 2:** Classification of reads from viromes hitting viral sequences. ssDNA: single-stranded DNA; dsDNA: double-stranded DNA.

Group	Order	Family	Relative Abundance (%)		
			D39s	DA4s	DA4b
dsDNA	*Caudovirales*	*Podoviridae*	41.92	48.70	42.15
	*Caudovirales*	*Myoviridae*	28.34	22.92	29.46
	*Caudovirales*	*Siphoviridae*	11.92	13.38	14.08
	*Caudovirales*	unclassified	3.56	2.99	2.90
	*Caudovirales*	*Phycodnaviridae*	3.57	2.22	1.32
	--	*Mimiviridae*	0.16	0.22	0.10
	--	*Poxviridae*	0.14	0.22	1.13
	--	*Iridoviridae*	0.37	0.44	0.30
	unclassified		3.13	2.84	3.17
ssDNA	--	*Inoviridae*	0.10	0.13	0.12
	--	*Microviridae*	0.00	0.10	0.04
	--	*Circoviridae*	0.01	0.11	0.01
	--	unclassified	0.09	0.05	0.05
virophage	--		2.00	0.58	0.19
others	*Ortervirales*	*Retroviridae*	0.26	0.29	0.21
	*Ortervirales*	*Caulimoviridae*	0.07	0.76	0.04
Unclassified phage/viruses	--		3.71	3.01	4.07

**Table 3:** Taxonomic characterization of South Scotia Ridge (SSR) viromes and twenty previously published viromes as determined by the indicated BLAST comparison to viral Refseq (*E*-value < 10^−3^).

Virome	MetaVir ID.	Total Sequenced Reads	Viral Hits (%)	Relative Abundance (%)																					
				dsDNA Viruses, No RNA Stage													RNA	ssDNA						Satellite	Virophage
				*Ackermann*	*Baculo*	*Myo*	*Podo*	*Sipho*	*Herpes*	*Irido*	*Mimi*	*Papilloma*	*Phycodn*	*Polyom*	*Pox*	Others		*Circo*	*Gemini*	*Ino*	*Micro*	*Nano*	Others		
Antar Lake Spring	10	41,322	20.51	0	0	0.05	0.06	0.22	0	0	0	0	0.01	0	0	0.03	0.14	22.00	0.27	0.03	3.45	0.22	23.39	6.82	0
Antar Lake Summer	11	38,475	22.07	0	0.01	0.32	0.30	0.72	0.03	0.01	0.38	0	1.57	0.02	0.09	0.20	0.40	61.96	0.49	0.11	1.31	0.93	15.52	3.20	0.19
Lake Pavin	6	649,290	25.04	0	0	1.60	1.29	6.73	0.01	0.01	0.01	0	0.06	0.01	0.02	0.85	28.04	6.02	0.07	0.12	1.79	0.06	17.07	0.93	0.25
Lake Bourget	7	593,084	37.46	0.01	0	2.30	2.98	11.56	0.02	0.01	0.01	0	0.14	0.02	0.06	1.86	0.28	1.77	0.03	0.01	60.35	0.32	1.99	0.36	0.20
Tilapia Channel	33	264,844	9.18	0.03	0.02	5.40	11.42	11.84	0.04	0.03	0.04	0	0.09	0.06	0	2.77	0.02	19.89	13.46	0.42	8.26	0.16	12.31	3.80	0.03
Lough Neagh	4925	2,295,055	24.65	0.14	0.08	10.96	32.59	36.71	0.11	0.08	0.02	0.06	0.38	0.05	0.31	16.11	0.32	0.01	0.03	0.16	0.04	0	0.39	0	0.13
Jiulong River Estuary	6305	498,957	31.40	0.05	0.06	14.67	32.36	34.80	0.12	0.04	0.11	0.02	0.79	0.16	0.49	13.58	0.27	0	0.01	0.11	0.01	0.01	0.22	0.31	0.14
GS117	1479	480,375	38.80	0.04	0.03	16.50	35.59	27.05	0.06	0.04	0.06	0.01	0.53	0.06	0.44	17.59	0.17	0.01	0.01	0.02	0.01	0.01	0.07	0.32	0.28
M1CS	1440	303,519	32.93	0.05	0.03	10.92	28.08	48.75	0.09	0.06	0.06	0.02	0.47	0.07	0.15	9.85	0.11	0.03	0.01	0.01	0	0.04	0.11	0.29	0.11
Dunk Island	1357	1,165,256	3.61	0.01	0.02	12.61	40.74	21.80	0.19	0.04	0.04	0.06	0.36	0.04	0.15	22.43	0.09	0	0.05	0.02	0	0	0.07	0.53	0.20
Fitzroy Island	1358	82,739	32.66	0.02	0.05	13.33	48.00	17.30	0.04	0.05	0.05	0.06	0.44	0.12	0.01	19.36	0.20	0	0.04	0.04	0	0	0.09	0	0.29
LA26S	1396	165,256	28.45	0.04	0.17	15.51	44.05	24.32	0.08	0.10	0.11	0.02	1.17	0.27	0.27	11.76	0.10	0	0.02	0.07	0	0	0.21	0.76	0.19
D39s	--	17,667,185	11.97	0.09	0.06	28.33	41.90	11.92	0.03	0.33	0.16	0	3.57	0.05	0.14	7.04	0.04	0.01	0	0.07	0	0	0.10	0	2.00
DA4s	--	19,101,812	5.09	0.06	0.22	22.91	48.68	13.37	0.05	0.41	0.22	0	2.21	0.06	0.22	6.33	0.10	0.11	0	0.10	0.10	0	0.07	0	0.58
DA4b	--	18,192,635	13.87	0.10	0.05	29.46	42.14	14.08	0.02	0.26	0.10	0	1.32	0.01	1.13	6.44	0.05	0.01	0	0.09	0.04	0	0.07	0	0.19
Sargasso Sea	12	397,939	11.80	0.01	0	2.20	6.32	2.48	0	0.02	0.02	0	0.08	0.04	0	2.88	0.01	14.21	0	0	43.69	0.03	24.83	0.60	0.01
Arctic Vir	15	686,209	1.30	0.04	0.04	9.91	9.63	23.14	0.54	0.08	0.25	0.05	1.24	1.37	0.76	3.19	0.99	0.64	0.04	5.18	0	0	1.88	16.07	0.06
Arctic Ocean	1158	79,646	14.15	0	0	0.12	0.12	0.39	0	0	0.01	0	0.03	0.01	0.02	0.09	0.32	63.14	0.09	0.16	2.17	0.19	13.93	4.25	0
Black Sea	1155	78436	16.47	0	0	0.05	0.03	0.08	0	0	0	0	0.02	0	0.01	0.02	0.12	56.33	0.60	0.01	0.88	0.17	14.24	5.79	0
Med Sea	1161	65,340	16.13	0	0.01	2.29	0.82	2.49	0.04	0.05	0.02	0.01	0.16	0.04	1.58	0.35	0.29	21.05	0	0	59.09	0	2.96	7.51	0
OMZst3viral10m	897	128,441	15.56	0.01	0.01	2.90	4.00	2.12	0.01	0.02	0.04	0	0.40	0.03	0.06	1.07	0.08	60.79	0	0.03	6.49	0.13	14.89	1.88	0.16
Antar hypolith	2726	1,057,535	12.74	0.01	0.01	1.31	1.47	7.15	0.13	0.01	0	0.05	0.02	0.03	0.02	0.36	0.13	0.03	0	0.21	88.42	0	0.25	0.09	0
Antar open soil	2727	870,687	16.99	0.08	0.05	9.25	4.96	16.74	0.29	0.05	0.03	0.06	0.24	0.05	0.09	3.10	0.43	0.01	0	0.03	63.28	0	0.40	0.07	0

**Table 4:** The percentage of reads in other published viromes obtained from MetaVir with a significant similarity (BLASTN, *E*-value < 10^−3^) to the SSR viromes.

Biome	Virome	MetaVir Project ID.	Number of Reads	South Scotia Sea		
				D39s	DA4s	DA4b
Antarctic seawater	D39s	-	35,334,370	100%	50.35%	49.60%
Antarctic seawater	DA4s	-	36,385,270	48.90%	100%	28.37%
Antarctic seawater	DA4b	-	38,203,624	45.88%	27.02%	100%
Seawater	OMZst3viral10m	897	128,441	6.50%	6.86%	10.69%
Seawater	GS117	1479	480,375	8.61%	9.09%	14.57%
Arctic seawater	Arctic Vir	15	686,209	1.45%	1.33%	2.10%
Seawater	Sargasso Sea	12	397,939	4.68%	4.97%	7.69%
POV seawater	Dunk Island	1357	1,165,256	0.55%	0.71%	1.14%
POV seawater	Fitzroy Island	1358	82,739	7.44%	9.11%	14.04%
POV seawater	LA26S	1396	165,256	16.75%	14.72%	21.02%
POV seawater	M1CS	1440	303,519	14.77%	15.59%	23.12%
Deep Ocean	**Arctic Ocean**	1158	79,646	1.64%	2.17%	4.22%
Deep Ocean	**Black Sea**	1155	78,436	0.57%	0.62%	0.69%
Deep Ocean	**Med Sea**	1161	65,340	0.86%	0.89%	2.04%
Freshwater	Lake Bourget	7	593,084	0.94%	0.87%	1.66%
Freshwater	Lake Pavin	6	649,290	0.25%	0.23%	0.44%
Antarctic freshwater	**Antar Lake Spring**	10	41,322	0.07%	0.06%	0.16%
Antarctic freshwater	**Antar Lake Summer**	11	38,475	0.41%	0.43%	1.87%
Freshwater	Lough Neagh	4925	2,295,055	0.31%	0.30%	0.52%
Freshwater	Jiulong River Estuary	6305	498,957	5.74%	6.06%	9.61%
Freshwater	**Tilapia Channel**	33	264,844	0.14%	0.15%	0.31%
Antarctic soil	**Antar open soil**	2727	870,687	0.43%	0.42%	0.59%
Antarctic hypolith	**Antar hypolith**	2726	1,057,535	0.26%	0.42%	0.36%

## Supplementary Materials

The following are available online at http://www.mdpi.com/1999-4915/11/2/95/s1, Figure S1: Map of area around the South Scotia Ridge (SSR) indicating where samples for metagenomic analysis were collected; Figure S2: Krona chart representing taxonomic composition of the sequence reads in the D39s from SSR surface seawater. Relative abundance of the sequence reads classified by the taxonomic grouping based on BLASTX similarity search (*E*-value <10^−3^); Figure S3: Krona chart representing taxonomic composition of the sequence reads in the DA4s from SSR surface seawater; Figure S4: Krona chart representing taxonomic composition of the sequence reads in the D39s from SSR bottom seawater; Figure S5: Comparison of viromes between SSR area and other environmental viromes depending on known taxonomic composition (stress value 6.76%); Figure S6: Top 10 relative abundance of viral species in the three SSR virome; Table S1: Details of the sampling sites where the viral metagenomes have been collected in the South Scotia Ridge; Table S2: General statistics of the gene annotation of the three SSR viromes; Table S3: Most represented viral genotypes among the viral hits according to complete normalized viral genome length and the total number of reads sequenced.
