# Supplementary material for: Metagenomic Characterization of the Viral Community of the South Scotia Ridge
Source: Viruses. 2019 Jan 24;11(2):95. doi: 10.3390/v11020095 (PMC6410227; doi:10.3390/v11020095)
Supplement: Supplementary file 1 [file viruses-11-00095-s001.pdf]

Table S1. Details of the sampling sites where the viral metagenomes have been collected in the South Scotia Ridge

Parameter	D39s	DA4s	DA4b
**Sampling data**	31 Dec 2016	28 Dec 2016	28 Dec 2016
**Sample volume**	300L	200L	200L
**Latitude**	60°59′S	61°19′S	61°19′
**Longitude**	48°47′W	54°7′W	54°7′
**Depth**	0m	0m	521m
**Salinity (‰)**	34.37	34.42	34.5
**Temperature**	-0.04℃	-0.15℃	-0.57℃

Table S2. General statistics of the genes annotation of the three SSR viromes

IMG genomes / Genes	3300028548(D39s)		3300028550(DA4s)		3300028925(DA4b)	
	Number	% of Assembled	Number	% of Assembled	Number	% of Assembled
**RNA genes**	3842	0.85	3235	0.94	5295	0.67
rRNA genes	556	0.12	460	0.13	449	0.06
5S rRNA	146	0.03	64	0.02	94	0.01
16S rRNA	84	0.02	79	0.02	98	0.01
18S rRNA	105	0.02	111	0.03	49	0.01
23S rRNA	90	0.02	81	0.02	128	0.02
28S rRNA	131	0.03	125	0.04	80	0.01
tRNA genes	3286	0.73	2775	0.81	4846	0.62
**Protein coding genes**	445825	99.15	340505	99.06	781899	99.33
Genes with Function Prediction	106282	23.64	63896	18.59	152327	19.35
COG Categories	76213	16.95	40835	11.88	104018	13.21
Pfam Categories	98599	21.93	57678	16.78	140226	17.81
KEGG Orthology	50973	11.34	26843	7.81	61686	7.84
Enzymes	31923	7.10	16046	4.67	38634	4.91
MetaCyc Pathways	18218	4.05	8893	2.59	19020	2.42
KEGG Categories	31249	6.95	16793	4.89	34757	4.42

COG, clusters of orthologous groups; KEGG, Kyoto Encyclopedia of Genes and Genomes.

Table S3. Most represented viral genotypes among the viral hits according to complete normalized by viral genomes length and the total number of reads sequenced.

Viral species	Relative abundance (%)			Host
	D39s	DA4s	DA4b	
***Prochlorococcus* phages**	3.34	2.89	3.68	cyanobacteria
***Synechococcus* phages**	12.05	9.93	13.68	cyanobacteria
**unclassified Cyanophage**	3.46	2.99	3.80	cyanobacteria
Puniceispirillum phage HMO-2011	20.70	25.74	18.49	SAR116
Pelagibacter phages	21.71	18.36	21.60	SAR11
Pseudomonas phages	4.16	4.13	4.70	*Pseudomonas*

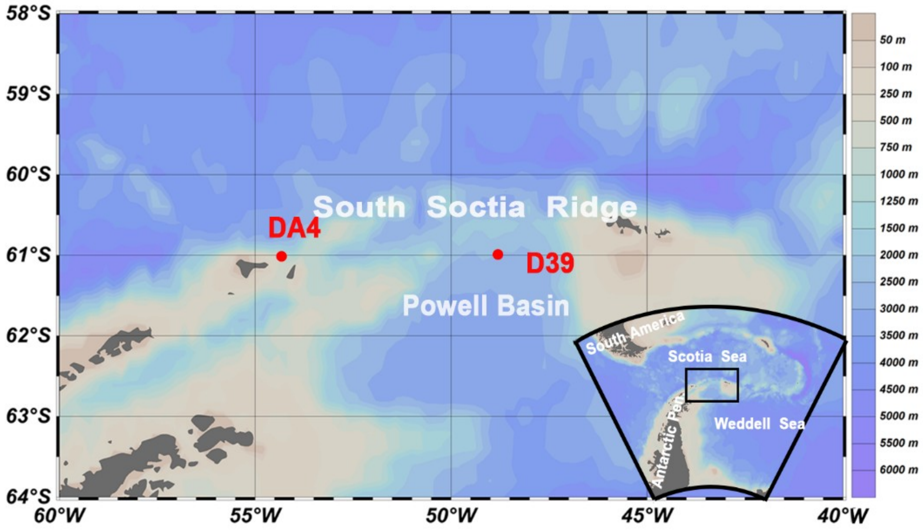

Fig. S1 Map of area around the South Scotia Ridge (SSR) indicating where samples for metagenomic analysis were collected.

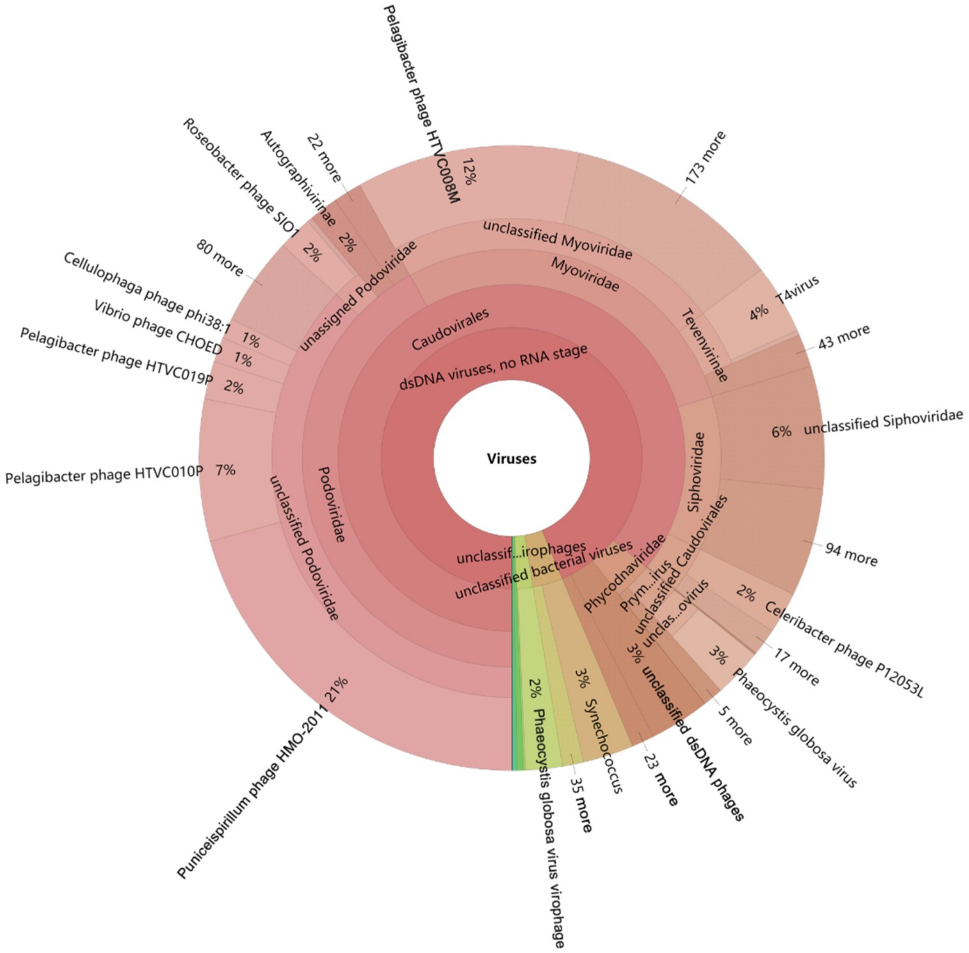

Fig. S2 Krona chart representing taxonomic composition of the sequence reads in the D39s from SSR surface seawater. Relative abundance of the sequence reads classified by the taxonomic grouping based on BLASTX similarity search (E-value < 10^{-3}).

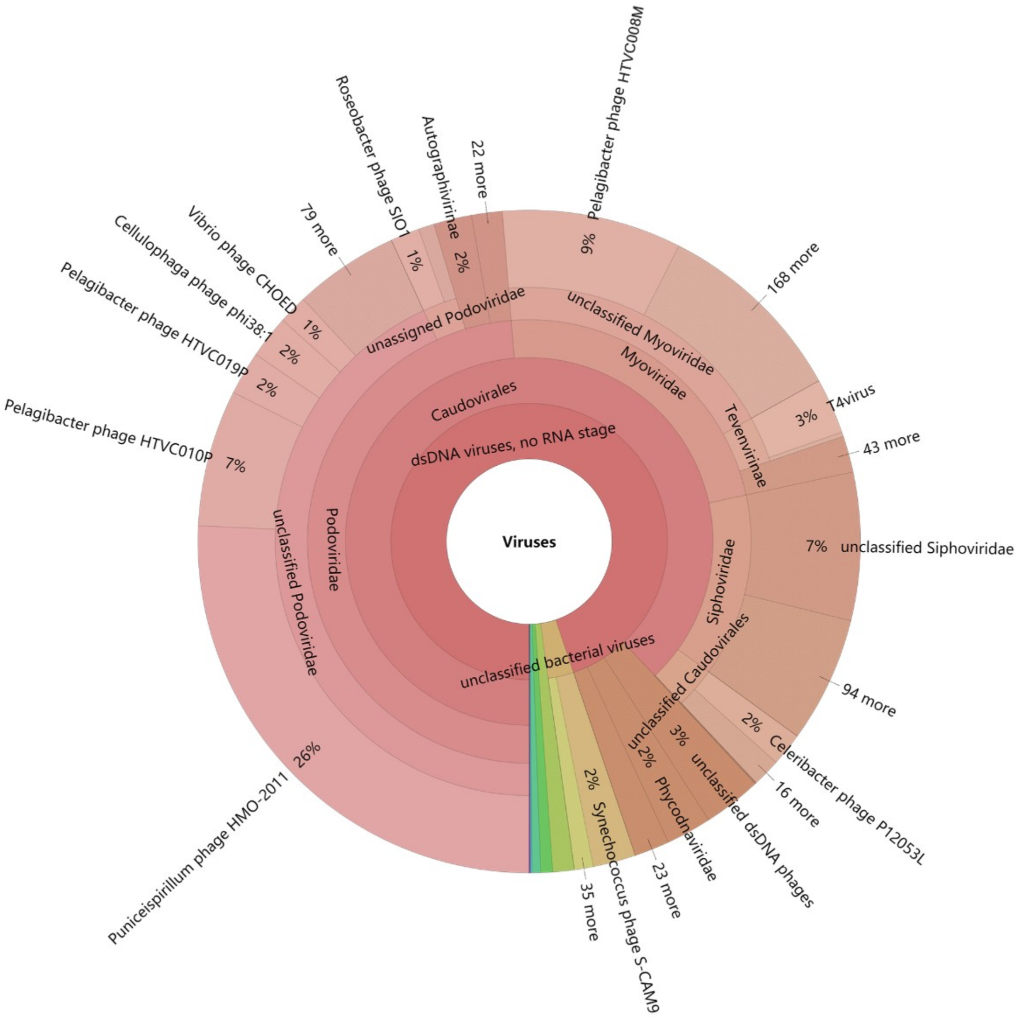

Fig. S3 Krona chart representing taxonomic composition of the sequence reads in the DA4s from SSR surface seawater.

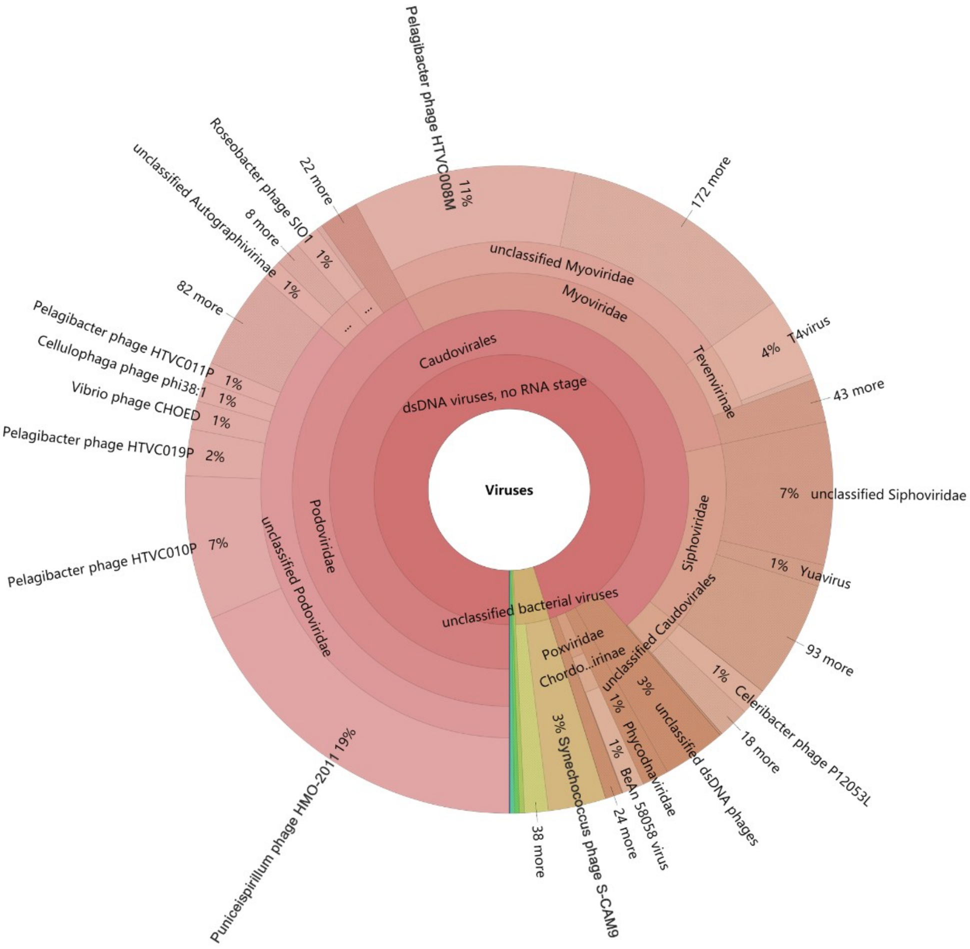

Fig. S4 Krona chart representing taxonomic composition of the sequence reads in the D39s from SSR bottom seawater

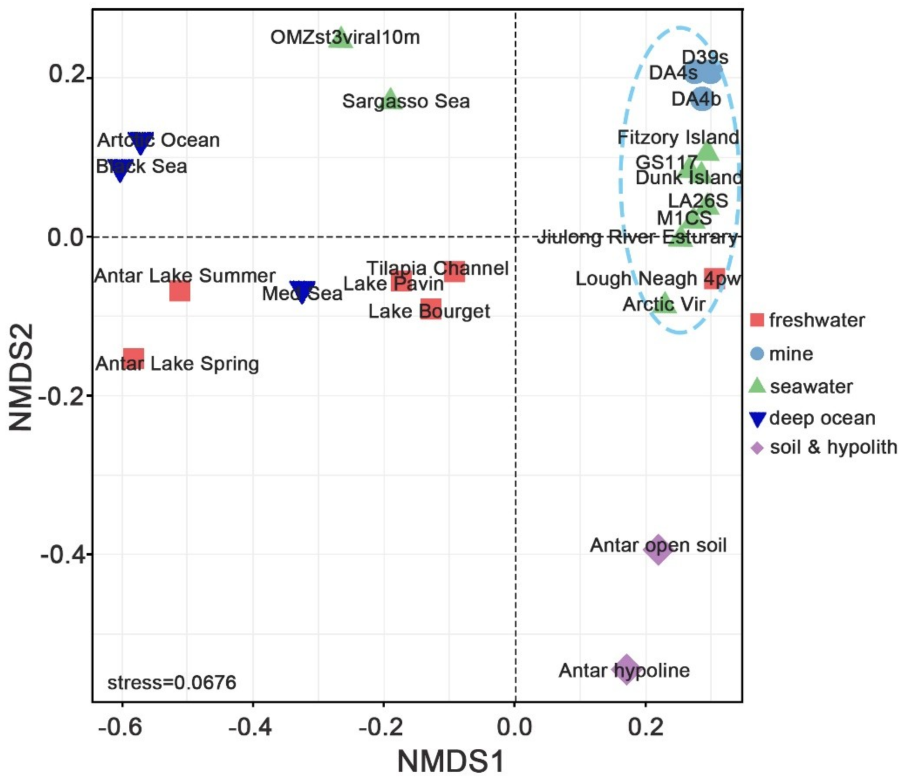

Fig. S5 Comparison viromes between SSR area and other environmental viromes depending on known taxonomic composition (stress value 6.76%), %)

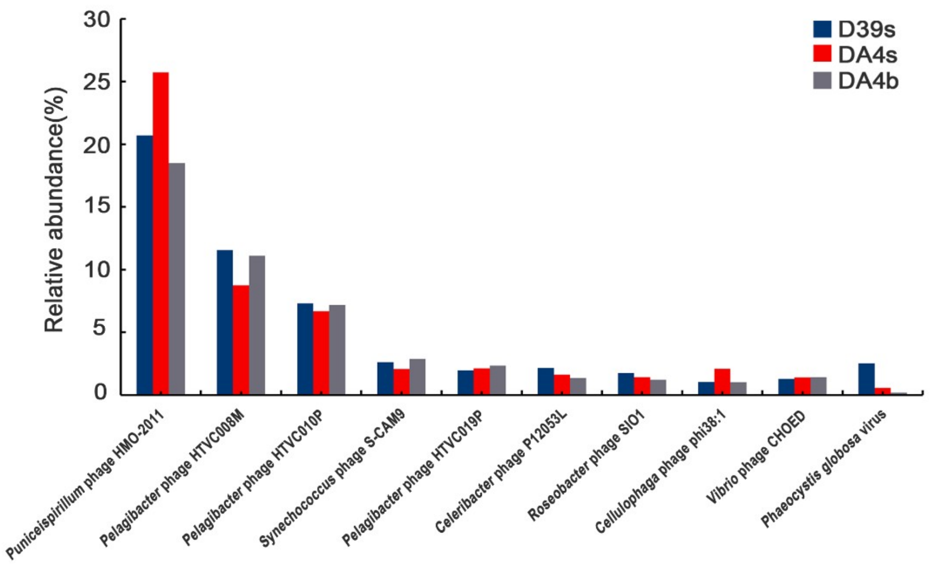

Fig. S6 Top 10 relative abundant of viral species in the three SSR viromes.
